# CMR Characteristics, gene variants and long-term outcome in patients with left ventricular non-compaction cardiomyopathy

**DOI:** 10.1186/s13244-021-01130-2

**Published:** 2021-12-11

**Authors:** Di Zhou, Shijie Li, Arlene Sirajuddin, Weichun Wu, Jinghan Huang, Xiaoxin Sun, Shihua Zhao, Jielin Pu, Minjie Lu

**Affiliations:** 1 Department of Magnetic Resonance Imaging, Fuwai Hospital and National Center for Cardiovascular Diseases, Chinese Academy of Medical Sciences, Peking Union Medical College, No. 167 Beilishi Road, Beijing, 100037 People’s Republic of China; 2 Department of Geriatrics, Beijing Friendship Hospital, Capital Medical University, Beijing, 100050 People’s Republic of China; 3 National Heart, Lung and Blood Institute (NHLBI), National, Institutes of Health (NIH), Bethesda, USA; 4 Department of Echocardiography, Fuwai Hospital and National Center for Cardiovascular Diseases, Chinese Academy of Medical Sciences, Peking Union Medical College, Beijing, People’s Republic of China; 5 Key Laboratory of Cardiovascular Imaging (Cultivation), Chinese Academy of Medical Sciences, Beijing, People’s Republic of China; 6 The Heart-Lung Testing Center, State Key Laboratory of Cardiovascular Disease, Fuwai Hospital, National Center for Cardiovascular Diseases, Chinese Academy of Medical Sciences, Peking Union Medical College, Beijing, People’s Republic of China; 7 Department of Nuclear Medicine, Fuwai Hospital and National Center for Cardiovascular Diseases, Chinese Academy of Medical Sciences, Peking Union Medical College, Beijing, 100037 People’s Republic of China; 8 Department of Cardiology, Shanghai East Hospital, Tongji University, Shanghai, People’s Republic of China

**Keywords:** Left ventricular non-compaction, Cardiomyopathy, Genetics, Cardiac magnetic resonance

## Abstract

**Background:**

As the paucity of data focusing on evaluating cardiac structure and function in patients with or without gene mutation, this study was sought to investigate the correlation between genotype and cardiac magnetic resonance (CMR) phenotype in patients with left ventricular non-compaction cardiomyopathy (LVNC) and to explore prognostic relevance in this cohort if possible.

**Methods:**

Patients with LVNC who underwent CMR and targeted gene sequencing between 2006 and 2016 were retrospectively evaluated. Demographic data, clinical presentation, genetic analysis, CMR data and follow-up data of all participants were collected.

**Results:**

Compared to negative genotype (G−) group, patients with positive genotype (G+) had larger left atrial volume (LAV), and carriers of multiple variants had lower left ventricular (LV) ejection fraction and cardiac index, increased LV fibrosis, larger LA volume, reduced LV global circumferential strain, LA reservoir strain and booster pump strain (all *p* < 0.05). LA volume was able to discriminate patients with G + (all *p* < 0.05), as well as those with multiple genetic mutation (all *p* < 0.01). During a median follow-up of 5.1 years, Kaplan–Meier survival analysis revealed worse primary endpoint-free survival among carriers of multiple variants compared to G− group.

**Conclusions:**

CMR feature tracking is a remarkable tool to evaluate implication, genetics cascade screen and predict outcome in LVNC population. LA volume is a sensitive and robust indicator for genetic mutational condition, of which facilities to guide clinical management and intensity of follow-up for patients and their relatives.

**Supplementary Information:**

The online version contains supplementary material available at 10.1186/s13244-021-01130-2.

## Key points

- CMR characteristics are associated with genetics, especially influenced by multiple genetic mutations interacting.
- LA volume is a sensitive and robust indicator of genetic mutational condition.
- CMR-FT is a remarkable tool to evaluate implication, genetics cascade screen and prognosis in LVNC population.

## Background

Left ventricular non-compaction cardiomyopathy (LVNC) is a genetically and clinically heterogeneous cardiomyopathy that can occur in isolation or in correlation with other congenital and acquired cardiac pathologies [1]. It is characterized by a heavily hypertrabeculated myocardium and deep intertrabecular recesses communicating with the left ventricular (LV) cavity and a thin epicardial compacted myocardium, morphologically reminiscent of early cardiac development [2, 3]. The abnormal morphology of LVNC is thought to be caused by the arrest of myocardial compaction during embryogenesis, characteristically in the final segments—the apical inferior and lateral segments, with variable basal extension [4, 5].

The American Heart Association classified LVNC as a genetic cardiomyopathy, and gene mutations are associated with those encoding sarcomere, Z disk, cytoskeletal, ion channel proteins, or mitochondrial, and those involved in cellular energy metabolism [6]. However, the European Society Association has defined LVNC as an “unclassified cardiomyopathy” that does not take a firm stance on whether it is a separate cardiomyopathy or a morphological trait [7]. And the results of studies regarding correlations between genotype and outcome are inconsistent [8–11]. One of the key issues underpinning this controversy is the paucity of data focusing on evaluating cardiac structure and function in patients with or without gene mutation.

Currently, echocardiography is considered to be the first line image modality for diagnosing LVNC with three different diagnostic criteria [12, 13], and cardiac magnetic resonance (CMR) is generally considered as a complementary tool for the diagnosis of LVNC or when the patient’s acoustics is poor or the diagnosis is undetermined by echo [4, 13–15]. However, due to the wide spectrum of clinical manifestations in patients with LVNC, ranging from no symptoms to arrhythmias, thromboembolic events, chronic heart failure, or sudden cardiac death, there is no universally definition of the severity of LVNC by imaging methods, let alone the association between imaging performance and genotype status [16]. Advances in technologies enable improved assessment of left heart phasic function (strain). Strain imaging detects the percentage change of myocardial deformation using echocardiography initially. Cardiac magnetic resonance feature tracking (CMR-FT) is a novel offline approach to assess myocardial deformation by the tracking of tissue voxel motion [17]. In view of the complicated myocardial disorders in LVNC, CMR-FT has some potential advantages that may perform a more accurate assessment considering view fields, spatial resolution and reproducibility [18]. In this study, we aim to investigate the genotype-CMR phenotype correlation in patients with LVNC and to explore prognostic relevance in this cohort if possible.

## Methods

### Study population

Patients with LVNC who underwent CMR at our Hospital between April 2006 and July 2016 were enrolled in the study. Diagnosis of LVNC was referred to consensus of re-evaluated echocardiography and CMR imaging, according to the Jenni and Petersen criteria by experienced cardiologist and radiologist [13, 15]. The exclusion criteria include the following: (a) age < 18 years, (b) CMR studies with limited image quality, (c) incomplete cine balanced steady-state free precession (bSSFP) coverage of the LV, (d) history of ischemic heart disease, (e) with others concomitant cardiomyopathy. Demographic data, clinical presentation and genetic analysis of all participants were collected. This study complies with the principles of the Declaration of Helsinki and was approved by the Ethics Committee of our hospital.

### Targeted sequencing

Genomic DNA was extracted from peripheral venous blood of each subject. The coding exons and their adjacent 10-bp intronic sequences of 72 cardiomyopathy-related genes were enriched referring to a custom-designed library (Agilent Technologies, Santa Clara, CA) and were screened on an Illumina next-generation sequencing platform. The mean depths of all samples were > 4009, with coverage of > 99.7%. Related details are described in Additional file 1: Data S1.

Variants were explained in the guidelines for mutation nomenclature of the Human Genome Variation Society. Variants were defined as polymorphisms and excluded once their minor allele frequency was ≥ 0.05% among East Asians in the Genome Aggregation Database [19]. The pathogenicity of detected variants was determined according to the recommendations of the American College of Medical Genetics and Genomics and the Association for Molecular Pathology, which was classified as “pathogenic,” “likely pathogenic,” “uncertain significance,” “likely benign,” or “benign” [19] Criteria for classification are described in Additional file 1: Tables S1 and S2. In this study, variants which were classified as pathogenic or likely pathogenic were considered to be pathogenic and accordingly grouped into genotype positive (G+) or genotype negative (G−), respectively. Moreover, Sanger sequencing was used to validate pathogenic variants. The primers, used for sequencing, are listed in Additional file 1: Table S3.

### CMR protocols

CMR images were performed at 1.5T MR (Magnetom Avanto; Siemens, Erlangen, Germany) with retrospective electrocardiogram gating and 8-channel cardiac coil. For morphologic, functional and subsequent feature tracking analysis, bSSFP breath-held cine images were acquired in the following planes: one 2-chamber view, one 3-chamber, one 4-chamber view, and 8 equidistant short-axis planes covering entire left ventricle. Conventional imaging parameters included the following: repetition time 2.9–3.4 ms, echo time 1.1–1.5 ms, field of view 320 × 320 mm ~ 380 × 380 mm, temporal resolution: 30–55 ms, slice thickness 8 mm, matrix size 192 × 162 [20]. Using a gradient spoiled fast low-angle shot sequence with phase-sensitive inversion recovery technique, late gadolinium enhancement (LGE) CMR imaging was performed in a series of sequential 6-mm LV short axis slices to cover the entire left ventricle. Myocardial LGE was identified by magnetic resonance images when 0.2 mmol/kg contrast agent has administrated for 10 min. The inversion time was individually assessed per patient to null the myocardial signal.

### CMR analysis

**Fig. 1:**
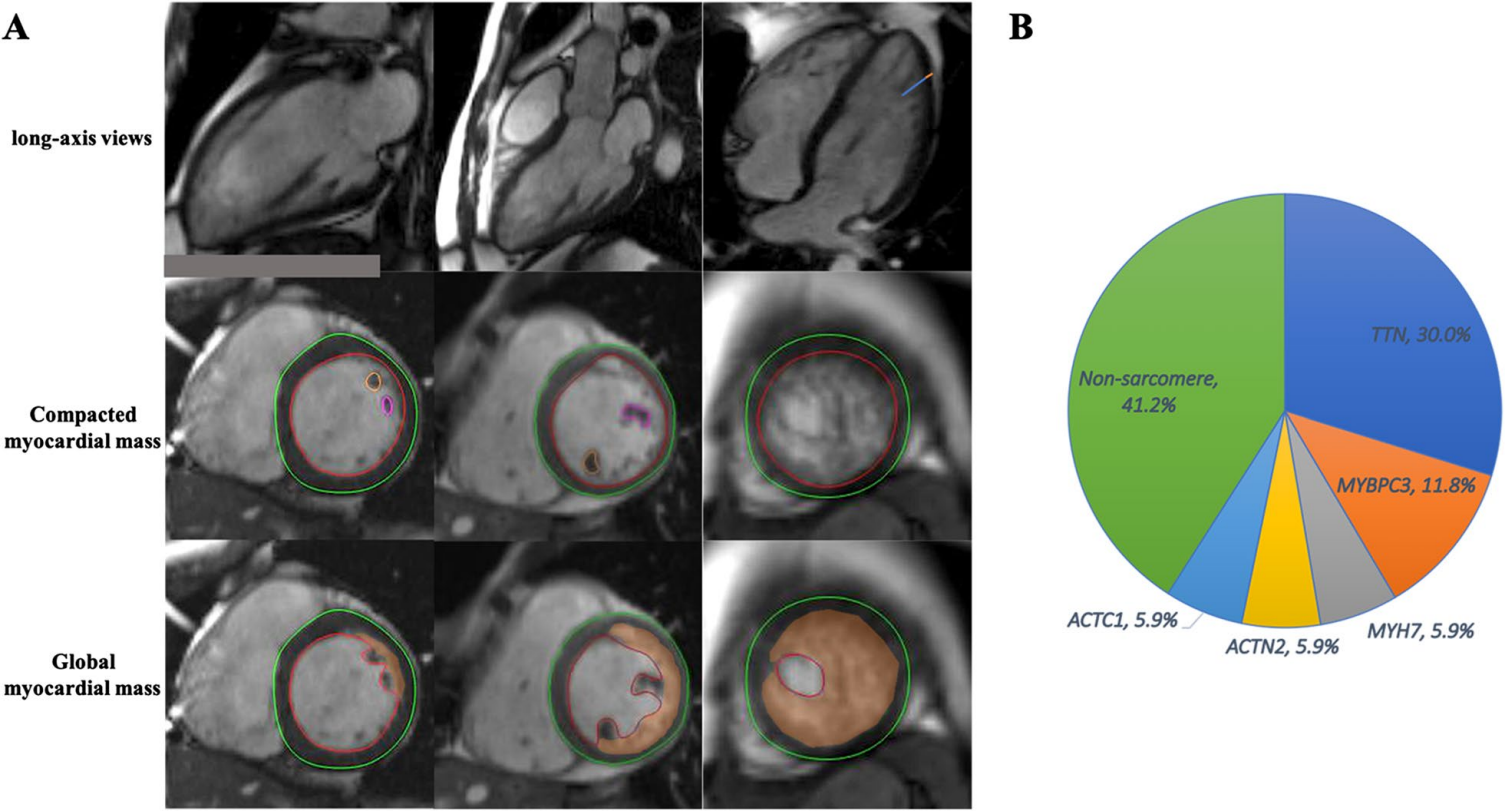
CMR characteristics and genetics in LVNC. **a** The orange line identifies the thickness of the compacted layer, and the blue line identifies the thickness of the trabeculated layer. Standard epicardial contours (green line) and endocardial contours (red line) for measurement of the compacted myocardial mass and the papillary muscles (yellow line, pink line) were included from the measurements. Endocardial contours (red line) at the border of the non-compacted myocardium for measurement of the ‘global myocardial mass.’ The non-compacted mass (orange region) was subsequently calculated by subtracting compacted mass and papillary muscle mass from global LV myocardial mass. **b** Spectrum of pathogenic variants in this cohort

LV volumes (LV end-diastolic volume index, LV stroke volume index, LV end-systolic volume index), LV cardiac index, LV ejection fraction (LVEF) and LV compacted mass index were measured using a workstation (Argus; Siemens Medical Solutions) based on the short-axis cine images. The normalized reference values of LV function metrics referred to Maceira AM criterion [21]. The non-compacted myocardium mass was assessed in accordance with previously validated method [22], which delineated the end-diastolic endocardial border of non-compacted myocardium along with conventional epicardial contours to calculate the indexed “global myocardial mass,” without accounting for the intertrabecular recesses of non-compacted myocardium (Fig. 1a). Note that papillary muscles were included in LV volume and compacted LA mass. Then, the indexed non-compacted mass was subsequently calculated by subtracting compacted mass index and papillary muscle mass index from indexed global LV myocardial mass, as well as their ratio. In addition, the extent of non-compacted and compacted LV thickness as well as their ratio were measured on one end-diastolic long axis geometry [15]. The quantitative extent of LV LGE was measured and expressed as a percentage of the LV mass. The LV myocardium was manually delineated by endocardial and epicardial contours. And then, LGE was semiautomatically determined by utilizing the full width at half maximum method and manual correction by using QMass (Medis Suite 3.1, The Netherlands) [23]. Any obvious pericardial partial volume artifacts and blood pool were manually corrected.

Left ventricular and atrial (LA) strain analysis were performed offline using a commercially available software (QStrain, Medis Suite 3.1, The Netherlands) based on 2-, 3- and 4-chamber and short axis cine images (Fig. 1) [24]. The software feature tracking performance was visually reviewed in order to ensure accurate tracking. In cases of insufficient tracking, the software allows for border readjusted and then propagation algorithm reapplied [25]. Radial and circumferential strain were obtained by standard endo- and epicardial contours to three selected slices at representative basal, mid-ventricular and apical levels in LV short axis cine bSSFP stack [26]. The selected basal slices showed circumferentially complete myocardium in the whole cardiac cycle, the selected mid-ventricular slices were at the level of the papillary muscles, and the selected apical slices excluded obliquely oriented myocardium. LV longitudinal strain was obtained by standard end-diastolic endocardial contours with the defining mitral valve plane and LV apex in LV 2-, 3- and 4-chamber cine bSSFP single-slice images. Global longitudinal strain (GLS), global circumferential strain (GCS), and global radial strain (GRS) were calculated from the average of the peak strain of the corresponding three slices. LA endocardial borders were manually drawn without pulmonary veins and the LA appendage which LA volume was obtained at late LV diastole after LA contraction (minimal LA volume, LAVmin), at LV diastole before LA contraction (LAVpre-a) and at LV end-systole (maximal LA volume, LAVmax). Three aspects of LA strain were calculated from outcomes as previously mentioned: total strain (εs, first strain peak, reflective of atrial reservoir function during LV systole), active strain (εa, second strain peak, reflective of LA booster pump function during late LV diastole) and passive strain (εe, difference between εs and εa, reflective of atrial conduit function during early LV diastole), which, respectively, correspond to LA reservoir function, boost pump function and conduit function [17, 27]. The normal values of myocardial strain have been reported in previous publications [28, 29].

### Follow-up

The primary endpoint was a composite of all-cause death, heart transplantation [9]. Patients were followed with telephone interviews by two independently trained investigators who used described criteria. The last follow-up was performed in April 2018. Medical records and copies of death certificates were requested to ascertain the incidence of clinical endpoints [30].

### Statistical analysis

Continuous variables were presented as the means ± standard deviation or medians and inter-quartile range, as appropriate. Categorical variables were expressed as numbers and percentages. Normally distributed continuous variables were verified using Kolmogorov–Smirnov test. Normally distributed and non-normally distributed continuous variables were compared using independent *t* test and Mann–Whitney *U*-test, respectively. Categorical variables were compared using Fisher’s exact test or *χ*^2^ test. Accordingly, Pearson or Spearman correlation was performed to investigate the correlation. The correlation was considered weak if *r* < 0.3, fair if r was between 0.3 and 0.5, moderate if r was between 0.5 and 0.7, and strong if *r* > 0.7 [31].

Receiver operating characteristic (ROC) curve was performed to identify the parameters with the highest sensitivity and specificity for discriminating genotype status. Univariable Cox regression models were computed to evaluate the unadjusted hazard of primary endpoint. Hazard ratios (HRs) with corresponding 95% confidence intervals were generated assessed with Durbin–Watson test. Moreover, the differences in event-free survival according to genotype status were evaluated by Kaplan–Meier survival analysis. Statistical analysis was performed by using IBM SPSS (version 22.0, Chicago). Statistical significance was defined as *p* < 0.05, which all values were 2 tailed.

## Results

### Study population and genetic profile

A total of 28 LVNC patients were ultimately included in the current study (mean age: 40.9 years ± 15.1), of 20 (71.4%) of them were male. 17 pathogenic variants were found in 11 (39.3%) of patients, of which 10 (58.8%) of variants were located in sarcomere genes (Additional file 1: Table S4). Among these genes, *TTN* was the most commonly involved gene (30%), followed by *MYBPC3* (11.8%), *KCNE1* (11.8%), *DMD* (11.8%), and *DSP* (11.8%) (Fig. 1b). Five patients were carriers of multiple (> 1) variants, and all of these carried both sarcomere and non-sarcomere genes in this study.

Arrhythmia was rather common and only three patients had a positive family history of LVNC. As shown in Table 1, there were no differences in clinical baseline characteristics between G + and G − group.

**Table 1:** Baseline clinical characteristics

Parameters	LVNC (*n* = 28)	G+ (*n* = 11)	G− (*n* = 17)	*p* value*
Age, years	40.9 ± 15.1	42.1 ± 10.4	40.1 ± 17.7	0.706
Male, *n* (%)	20 (71.4)	7 (63.6)	13 (76.5)	0.671
BSA, m^2^	1.8 ± 0.2	1.9 ± 0.3	1.7 ± 0.2	0.141
Smoking, *n* (%)	4 (14.3)	2 (18.2)	2 (11.8)	0.454
Drinking, *n* (%)	2 (7.1)	1 (9.1)	1 (5.9)	0.810
Hypertension, *n* (%)	3 (10.7)	0 (0)	3 (17.1)	0.258
Hypercholesterolemia, *n* (%)	7 (25)	5 (45.5)	2 (11.8)	0.076
NYHA 3/4, *n* (%)	7 (25)	4 (36.8)	3 (17.6)	0.381
Family history LVNC, *n* (%)	3 (10.7)	2 (18.2)	1 (5.9)	0.543
Arrhythmia, *n* (%)	22 (78.6)	9 (81.8)	13 (76.5)	> 0.99

Data are expressed as mean ± SD (standard deviation) or *n* (%)

*LVNC* left ventricular non-compaction cardiomyopathy, *G*+ genotype positive, *G*− genotype negative, *BSA* body surface area, *NYHA* New York Heart Association class

*Significance of difference between parameters from G+ and G− groups

### Cardiac magnetic resonance data

Overall, the ratio of non-compacted and compacted LV thickness was 2.9 (2.5–3.3) (Table 2). Patients with LVNC had significantly lower LVEF (34.2% ± 17.6) and LV stroke volume index (39.7 ml/m^2^ ± 17.6) as well as greater LV end-diastolic volume index (133.4 ml/m^2^ ± 52.3) and LV end-systolic volume index (93.7 ml/m^2^ ± 56.5) (Table 2). Impaired left ventricular and atrial strain were observed in all of subjects, but a partial of patients did not show significant enlargement of LA volume.

**Table 2:** Cardiac magnetic resonance data

Parameters	LVNC (*n* = 28)	G+ (*n* = 11)	G− (*n* = 17)	*p* value*
LVEF, %	34.2 ± 17.6	32.6 ± 17.0	35.2 ± 18.4	0.703**†**
LVEDVi, ml/m^2^	133.4 ± 52.3	124.5 ± 42.6	139.2 ± 58.3	0.479
LVESVi, ml/m^2^	93.7 ± 56.5	77.9 (59.0, 121.3)	67.5 (44.8, 146.3)	0.733
LVSVi, ml/m^2^	39.7 ± 17.6	35.4 ± 17.4	42.4 ± 17.7	0.832
LVCI, l/min·m^2^	2.3 (1.5, 2.9)	2.0 (1.5, 3.7)	2.8 (2.1, 3.8)	0.384**†**
LV-C, mm	4.9 ± 1.2	5.3 ± 1.4	4.6 ± 1.0	0.123
LV-NC, mm	14.4 ± 4.0	16.1 ± 4.6	13.4 ± 3.4	0.084
LV-NC/C	2.9 (2.5, 3.3)	2.8 (2.7, 3.2)	2.8 (2.5, 3.2)	0.944
LVMi-C, g/m^2^	64.5 ± 16.7	58.2 ± 13.8	68.6 ± 17.5	0.107
LVMi-NC, g/m^2^	63.9 ± 24.9	66.4 ± 25.5	62.2 ± 25.2	0.671
LVMi-MM, g/m^2^	128.4 ± 37.4	124.6 ± 33.8	130.9 ± 40.3	0.674
LVMi-NC/MM	0.5 ± 0.1	0.5 (0.5, 0.6)	0.5 (0.4, 0.5)	0.070
LGE, %	13.5 ± 11.2	17.3 ± 13.6	11.2 ± 9.4	0.258**†**
LGE, n (%)	19 (67.9)	7 (70)	12 (66.7)	> 0.99
*Myocardial deformation*				
LVGLS, %	− 12.8 ± 5.2	− 11.6 ± 3.9	− 13.5 ± 5.8	0.350
LVGCS, %	− 16.9 ± 9.5	− 13.5 (− 16.0, − 6.2)	− 22.4 (− 25.8, − 9.1)	0.466**†**
LVGRS, %	22.2 (12.6, 45.1)	28.4 ± 25.9	29.4 ± 19.2	0.910
LAVmax, ml	66.7 (45.2, 130.5)	116.7 ± 61.7	66.6 ± 37.8	0.029**†**
LAVpre-a, ml	51.4 (33.9, 102.4)	93.7 ± 54.4	52.4 ± 32.2	0.048†
LAVmin, ml	30.2 (18.2,92.5)	69.7 (25.8, 128.1)	22.7 (12.4, 61.9)	0.013†
LAEFtotal, %	43.9 (29.0, 64.4)	37.6 ± 17.4	51.3 ± 18.9	0.066**†**
LAEFpassive, %	22.0 ± 10.8	20.7 ± 9.7	22.8 ± 11.7	0.623
LAEFactive, %	24.3 (12.3, 51.5)	18.1 (11.4, 46.8)	34.8 (13.0, 59.1)	0.120**†**
*ε*s, %	21.8 (13.9, 42.8)	17.2 (12.6, 36.7)	31.4 (19.8, 47.2)	0.115**†**
*ε*e, %	12.6 ± 10.6	10.2 ± 6.6	14.2 ± 12.4	0.281
*ε*a, %	11.9 (5.6, 21.2)	12.5 ± 9.1	19.2 ± 13.8	0.134**†**

Data are expressed as mean ± SD (standard deviation) or median (inter-quartile range)

*LVNC* left ventricular non-compaction cardiomyopathy, *G*+ genotype positive, *G-* genotype negative, *LVEF* left ventricular ejection fraction, *LVEDVi* LV end-diastolic volume index, *LVESVi* LV end-systolic volume index, *LVSVi* LV stroke volume index, *LVCI* LV cardiac index, *C* compacted thickness, *NC* non-compacted thickness, *NC/C* the ratio of non-compacted and compacted myocardial thickness, *LVMi-C* LV end-diastolic compacted mass index, *LVMi-NC* LV end-diastolic indexed NC mass, *LVMi-MM* indexed global LV myocardial mass, *LVMi-NC/MM* the ratio of non-compacted and global myocardial mass, *LGE* late gadolinium enhancement, *GLS* global longitudinal strain, *GCS* global circumferential strain, *GRS* global radial strain, *LAVmax* maximal left atrial volume, *LAVpre-a* left atrial volume before LA contraction, *LAVmin* minimal left atrial volume, *LAEF* left atrial emptying fraction, *εs* total strain, *εe* passive strain, *εa* active strain

*Significance of difference between parameters from G+ and G− groups

^†^Significance of difference between parameters from carriers of multiple variants and G- group

As shown in Table 3, correlation analysis revealed that LV end-diastolic volume index was strongly associated with indexed left ventricular mass (all *p* < 0.001). LV global deformations were associated with CMR parameters regarding the non-compacted areas, such as LV non-compacted thickness, LV indexed non-compacted mass. There were fair correlations between LV strain and the thickness and mass index of compacted myocardial. There was nonsignificant trend toward LA parameters derived from CMR-FT and parameters of compacted and non-compacted areas.

**Table 3:** Correlations between cardiac magnetic resonance parameters

	LVEF		LVEDVi		GLS		GCS		GRS	
	*r*	*p* value	*r*	*p* value	*r*	*P* value	*r*	*p* value	*r*	*p* value
LV-C	− 0.367	0.163	0.271	0.085	0.481	0.002	0.551	0.125	− 0.297	0.083
LV-NC	− 0.559	0.002	0.636	< 0.001	0.620	< 0.001	0.719	< 0.001	− 0.518	0.005
LV-NC/C	− 0.329	0.088	0.465	0.013	0.222	0.256	0.305	0.115	− 0.328	0.088
LVMi-C	− 0.517	0.005	0.705	< 0.001	0.337	0.079	0.406	0.032	− 0.443	0.018
LVMi-NC	− 0.551	0.002	0.885	< 0.001	0.618	< 0.001	0.648	< 0.001	− 0.585	0.001
LVMi-MM	− 0.598	0.001	0.905	< 0.001	0.563	0.002	0.614	0.001	− 0.588	0.001
LVMi-NC/MM	− 0.256	0.189	0.515	0.005	0.503	0.006	0.456	0.015	− 0.310	0.108

*p* values for Pearson or Spearman correlation analysis

Abbreviation as in Table 2

### Correlation between genotype and CMR phenotype

**Fig. 2:**
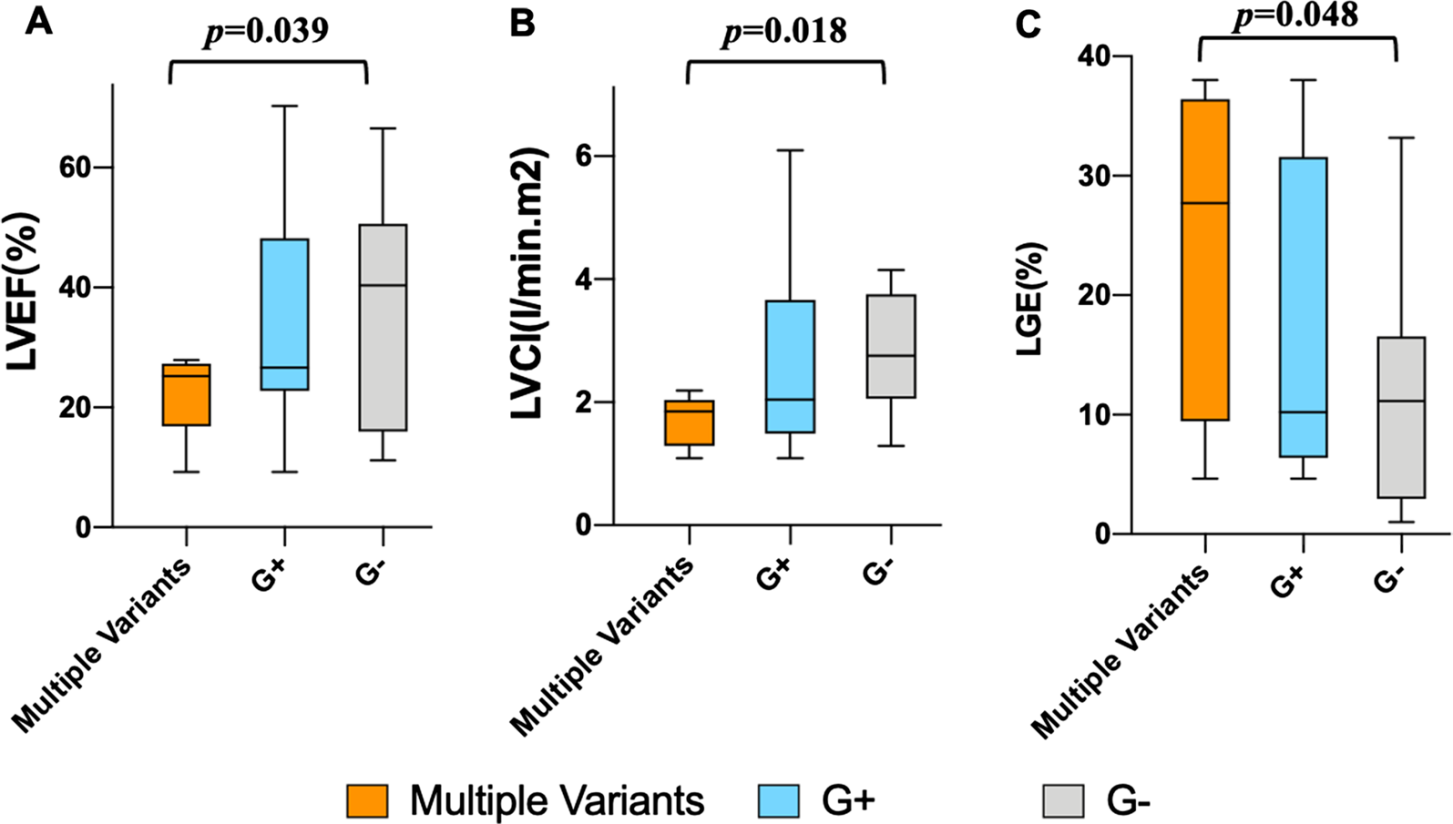
Correlations among carriers of multiple variants, G+ group and G− group in LVEF (**a**), LVCI (**b**) and LGE (**c**). *p* values for independent *t* test or Mann–Whitney U-test between parameters from carriers of multiple variants and G− group. *G*+ genotype positive, *G-* genotype negative, *LVEF* left ventricular ejection fraction, *LVCI* left ventricular cardiac index, *LGE* late gadolinium enhancement

There were no significant differences between G+ patients and G− patients in the LV volumetric and morphological parameters (Table 2). These two groups had a similar prevalence (*n* = 7, 70.0% vs. *n* = 12, 66.7%, *p* > 0.99) and percentage of LGE (17.3% ± 13.6 vs. 11.2% ± 9.4, *p* = 0.258) which only 7 patients involved in LGE > 15%. Lower LVEF, LV cardiac index and larger percentage of LGE were observed in carriers of multiple variants compared to noncarriers (*p* = 0.039, 0.018, and 0.048, respectively) (Fig. 2).

**Fig. 3:**
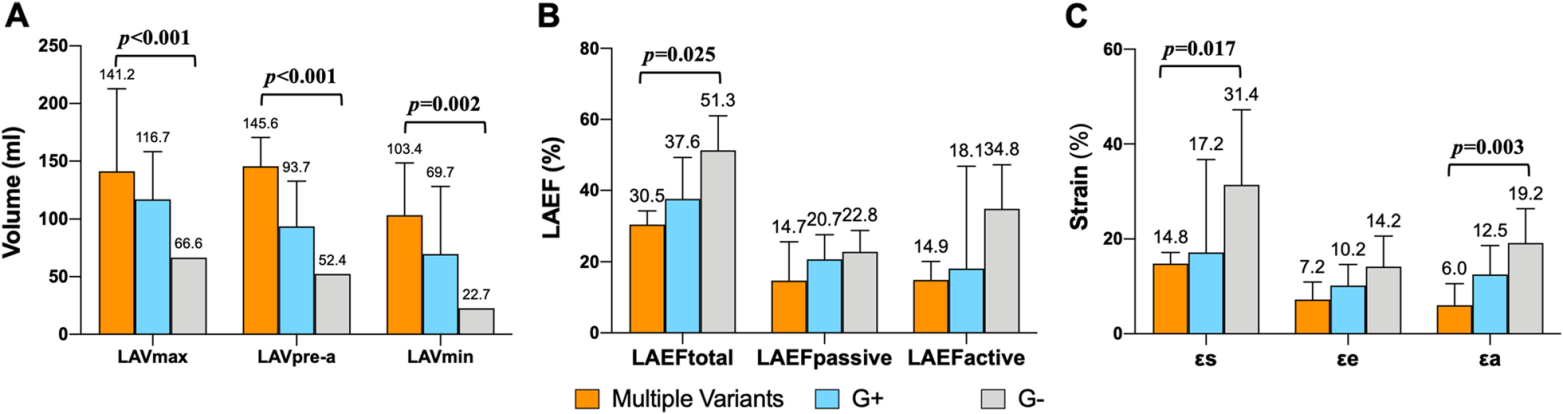
Correlations among carriers of multiple variants, G+ group and G− group in left atrial volume (**a**), left atrial emptying fraction (**b**), and left atrial longitudinal strain (**c**). *p* values for independent *t* test or Mann–Whitney *U*-test between parameters from carriers of multiple variants and G− group. *G*+ genotype positive, G− genotype negative, *LAVmax* maximal left atrial volume, *LAVpre-a* left atrial volume before LA contraction, *LAVmin* minimal left atrial volume, *LAEF* left atrial emptying fraction, *εs* total strain, *εe* passive strain, *εa* active strain

**Fig. 4:**
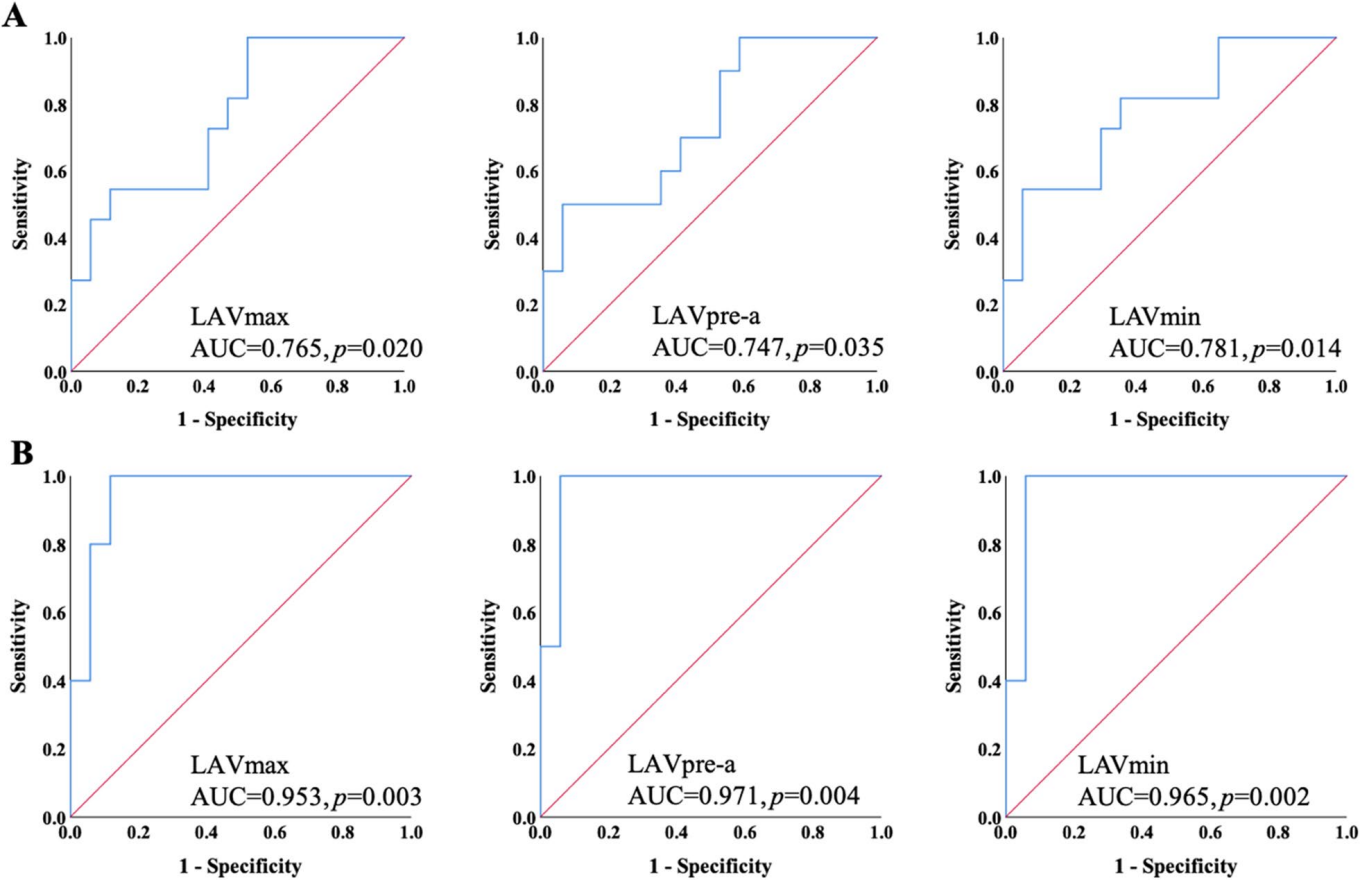
Receiver operating characteristic curve analysis for discriminating patients with positive genotype (**a**) and multiple genetic mutations (**b**). *AUC* area under the curve, *LAVmax* maximal left atrial volume, *LAVpre-a* left atrial volume before LA contraction, *LAVmin* minimal left atrial volume

CMR feature tracking analysis detected significantly increased LA volume in the G + groups, compared to G − cases (Table 2). No significant differences were noted in left ventricular and atrial deformation between G+ and G− groups. Additional file 1: Table S5 shows the results of regional LV strain. Compared to G− patients, carriers of multiple variants were associated with reduced LVGCS, increased LA volume, reduced LA strain and emptying fraction corresponding to reservoir and contractile function (Fig. 3). Using ROC analysis, LA volume was able to discriminate patients with G+ (all *p* < 0.05), and strongly predicted carriers of multiple variants in this cohort (all *p* < 0.01) (Fig. 4).

### Clinical outcomes

**Fig. 5:**
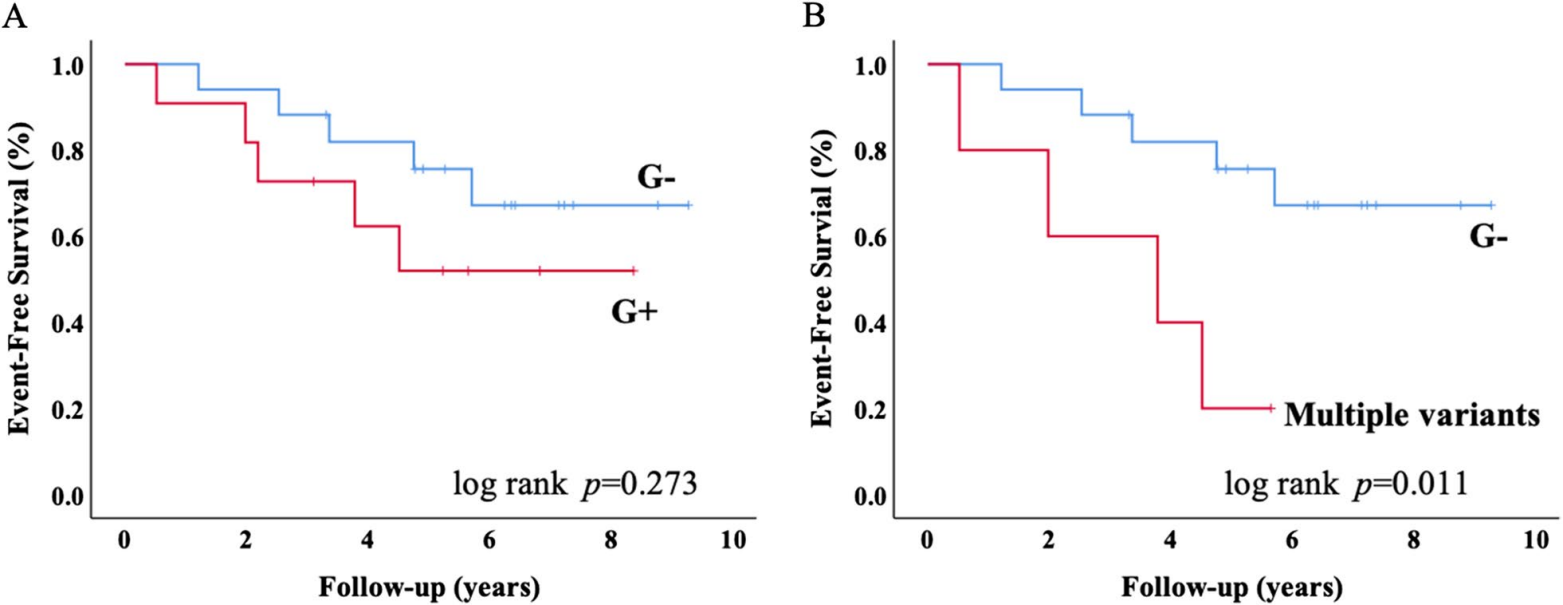
Kaplan–Meier survival curves for survival free of the primary endpoint according to positive genotype (**a**) and multiple genetic mutations (**b**). G+ genotype positive, G− genotype negative

During a median follow-up of 5.1 years (interquartile range, 3.3–6.8 years), 7 (23.3%) patients were documented cardiovascular death and 3 (10%) patients underwent from heart transplantation. Of those, 5 (50%) were G+ and 4 (40%) were carriers of multiple variants. No patients lost follow-up. Patients presented with adverse events were associated with lower EF, larger volume index and more impaired deformation in left ventricle and atria as well as LV indexed mass and fibrosis. Using univariate Cox analysis, variables as previously mentioned emerged as predictors of primary endpoint as well as LV regional strain (Table 4, Additional file 1: Table S6). Kaplan–Meier survival analysis revealed worse primary endpoint-free survival among carriers of multiple variants, whereas not of genotype positive (Fig. 5).

**Table 4:** Univariate Cox analysis for predicting primary endpoint during the follow-up

Parameters	LR *χ*^2^ (*p* value)	Wald	HR (95% CI)	*p* value
Age	7.627 (0.006)	6.759	1.064 (1.015–1.115)	0.006
Sex	0.427 (0.513)	0.418	0.599 (0.126–2.835)	0.518
Body surface area	0.359 (0.549)	0.357	0.436 (0.029–6.637)	0.550
Genotype status	1.202 (0.273)	1.158	1.982 (0.570–6.887)	0.282
LV-C	5.028 (0.025)	4.740	1.699 (1.054–2.739)	0.029
LV-NC/C	0.231 (0.631)	0.229	1.208 (0.557–2.618)	0.632
LVMi-C	8.532 (0.003)	7.571	1.049 (1.014–1.086)	0.006
LVMi-MM	11.950 (0.001)	9.987	1.025 (1.009–1.041)	0.002
LGE	4.726 (0.030)	4.267	1.056 (1.003–1.112)	0.039
LVEF	12.523 (< 0.001)	9.309	0.910 (0.859–0.967)	0.002
LVEDVi	8.035 (0.005)	6.999	1.011 (1.003–1.019)	0.008
Vmax	11.271 (0.001)	8.706	1.023 (1.008–1.038)	0.003
Vpre-a	11.560 (0.001)	8.858	1.024 (1.008–1.040)	0.003
Vmin	10.092 (0.001)	7.875	1.024 (1.007–1.040)	0.005
*ε*s	5.806 (0.016)	4.976	0.930 (0.872–0.991)	0.026
*ε*e	6.158 (0.013)	5.548	0.900 (0.824–0.982)	0.019
*ε*a	2.986 (0.084)	2.736	0.944 (0.882–1.011)	0.098
GLS	7.223 (0.007)	0.007	1.195 (1.044–1.368)	0.010
GCS	10.984 (0.001)	7.72	1.187 (1.052–1.339)	0.005
GRS	5.874 (0.015)	5.189	0.951 (0.911–0.993)	0.023

Abbreviation as in Table 2

## Discussion

In this single-center study, the correlation among CMR characteristics, genetic profile, and long-term outcomes in patients with LVNC was completely investigated. Key findings are as follows. First, in this heterogeneous cardiomyopathy, LV systolic dysfunction, LV fibrosis, LA enlargement and dysfunction, and risk of adverse events were linked to genetics, to some extent. Second, LA volume was strongly able to discriminate patients with G+ and carriers of multiple variants from G−. It is important to guide clinical management and intensity of follow-up for distinct groups of patients and their relatives. Finally, CMR metrics provided important prognostic information in patients with LVNC, whereas the complicate association between genetics and long-term outcome need to be explored in larger cohort furtherly.

Up-to-date, at least 21 genes have been identified, including sarcomeric, ion channel genes, and cytoskeletal, which are associated with LVNC. Despite differences in patient enrollment and gene panel used, the overall yield of our gene panel was 39.3% in accordance with a described yield of 35–40% [3]. In views of the advantage of quantifying LV morphology and function, Jacquier et al. [22] introduced a method for measuring LV trabeculation and permitting the diagnosis of LVNC using CMR. Global and regional LV strain reduction, derived by CMR-FT, has already been shown in children and adult cohorts, especially in mid-ventricular and apical segments mirrored manifested regions of the disease as well [26, 32]. Based on CMR imaging, van Waning et al. [8] suggested that the risk for LV systolic dysfunction (LVEF of < 45%) was associated with G+ status in patients with LVNC. Although there were no significant differences in LV structural, functional, global and regional deformation parameters between patients with and without gene mutation, we found lower LVEF, LV cardiac index, reduced GCS and increased myocardial fibrosis in carriers of multiple variants. Miszalski et al. [10] also demonstrated that strong correlations between mutational burden in individuals and LVEF, presence of LGE. Similar to the findings regarding LVNC and hypertrophic cardiomyopathy [10, 33], multiple mutations have been shown to elicit a severe phenotype that may act in a synergistic manner. The present findings, in part, help to close the knowledge gap regarding the clinical presentation heterogeneity in LVNC population. This also proposed that some cases may include hitherto unrecognized LVNC genes and variants, which alone are benign, all of which may impact the genetic landscape of LVNC.

Moreover, it has been demonstrated that impaired LA function is an independently predictor of new-onset atrial fibrillation. During hemodynamic stress or exertion, LA serves as the modulation of LV diastolic filling and cardiac performance by reservoir, conduit, and booster pump function [34]. In 339 long-term survival study of LVNC participants, LA dilation detected by echocardiography was present in 50% of patients, which was significantly associated with all-cause mortality (hazard ratio:3.20, *p* < 0.001). In our study, LA volumetric and deformation measurements were evaluated by CMR-FT for the first time. Compared to G−, patients with G+ had significantly larger LA volume, whereas not of myocardial strain parameters. And patients with multiple mutation had more severe LA reservoir (total LA emptying fraction, total strain) and booster pump (active strain) dysfunction. There is a subtype, restrictive LVNC, characterized by LA or biatrial dilation and diastolic dysfunction [3]. A research consisted of 232 children reported that this phenotype affected patients on poor outcomes, typically due to arrhythmia-related sudden cardiac [35]. This may explain the correlation between genetics and the presence of arrhythmia burden. In addition, based on ROC analysis, increased LA volume could significantly predict positive genetic status as well as multiple mutation. Our discovery is of great importance, which LA enlargement has promising utility to screen individuals with high risk of mutation and recommend DNA testing, in relatively cost-effective method. In this way, identifying the pathogenic mutation contributes to genetic cascade screen and fulfill regular cardiac follow-up in their offspring.

It is well recognized that 15–30% of patients with LVNC could suffer from premature death, and approximately 10% could develop severe HF that may eventually lead to HT [36]. One more finding in this study is that metrics derived from CMR-FT had great prognostic capable in LVNC population. We found that volumetric parameters of left atrial (LA maximal, diastasis, and minimal volume) and myocardial global strain (LAεs, LAεe, LAεa, LVGLS, LVGCS, LVGRS), LGE and LVEF were significantly associated with the primary endpoint without the ratio of non-compacted and compacted LV thickness (LV-NC/C) and the ratio of non-compacted and global myocardial mass (LVMi-NC/MM) in diagnostic criteria. Prior research has reported that LVNC patients detected pathogenic variants had higher risks of death and HT independent to other prognostic factors in a larger cohort study [9]. Although there was no statistical significance between G+ and G− groups in this cohort, carriers with multiple variants were associated with poor outcomes, along with more advanced CMR presentation.

There were several limitations in this study. First, this was an observational study, which might have been associated with an intrinsic bias. Second, this is a single-center study with relatively small sample size, which might limit the generalizability of the findings and need larger populations to confirm these finding, especially in prognostic result. Third, differences of strain measurements caused by various CMR-FT vendors cannot be excluded. So standardized postprocessing methods would be desirable to facilitate comparative analysis of LV deformation and reduce inter-vendor variability.

## Conclusions

Cardiac involvement, assessed by CMR presentation, is associated with genetic status in patients with LVNC, especially influenced by multiple genetic mutations interacting. LA volume is a sensitive and robust indicator of genetic mutational condition, of which facilities to guide clinical management and intensity of follow-up for patients and their relatives. CMR-FT is a remarkable tool to evaluate implication, genetics cascade screen and provide prognostic information in LVNC population.

## Supplementary Information

**Additional file 1**. **Data S1.** Gene panel. **Table S1.** Criteria for classifying pathogenic variants according to ACMG guideline. **Table S2.** Rules for combining criteria for pathogenic and likely pathogenic variants. **Table S3.** Primers for Sanger sequencing confirmation. **Table S4.** Pathogenic and likely pathogenic variants detected in the cohort. **Table S5.** Left ventricular segmental strain data assessed by CMR feature tracking. **Table S6.** Univariate Cox analysis of LV segmental strain for predicting primary endpoint.

## Data Availability

The datasets used and/or analyzed during the current study are available from the corresponding author on reasonable request.

## Abbreviations

AUC: Area under the curve
bSSFP: Balanced steady-state free precession
CMR: Cardiac magnetic resonance
CMR-FT: CMR feature tracking
G −: Genotype negative
G +: Genotype positive
GCS: Global circumferential strain
GLS: Global longitudinal strain
GRS: Global radial strain
LA: Left atrial
LAVmax: Maximal left atrial volume
LAVmin: Minimal left atrial volume
LAVpre-a: Left atrial volume before LA contraction
LGE: Late gadolinium enhancement
LV: Left ventricular
LVEF: Left ventricular ejection fraction
LVNC: Left ventricular non-compaction cardiomyopathy
ROC: Receiver operating characteristic
