# Supplementary material for: CMR Characteristics, gene variants and long-term outcome in patients with left ventricular non-compaction cardiomyopathy
Source: Insights Imaging. 2021 Dec 11;12:184. doi: 10.1186/s13244-021-01130-2 (PMC8665949; doi:10.1186/s13244-021-01130-2)
Supplement: Supplementary file 1 — Additional file 1. Data S1. Gene panel. Table S1. Criteria for classifying pathogenic variants according to ACMG guideline. Table S2. Rules for combining criteria for pathogenic and likely pathogenic variants. Table S3. Primers for Sanger sequencing confirmation. Table S4. Pathogenic and likely pathogenic variants detected in the cohort. Table S5. Left ventricular segmental strain data assessed by CMR feature tracking. Table S6. Univariate Cox analysis of LV segmental strain for predicting primary endpoint. [file 13244_2021_1130_MOESM1_ESM.docx]

**ELECTRONIC SUPPLEMENTARY MATERIAL**

**Data S1. Gene panel**

We designed a panel of 72 gene which have been implicated in LVNC, other cardiomyopathies or ion channel disease. The panel consists of the following genes: *ABCC9, ACTC1, ACTN2, BAG3, CALR3, CASQ2, CAV3, CFL1, CFL2, CMYA5, CRIP2, CRYAB, DES, DMD, DMPK, DSC2, DSG2, DSP, DTNA, FHL1, FHL2, FLNC, GPD1L, HCN4, JPH2, JUP, KCNE1, KCNE2, KCNE3, KCNH2, KCNJ2, KCNQ1, LAMP2, LDB3, LMNA, MIB1, MYBPC3, MYH6, MYH7, MYL2, MYLK3, MYOM1, MYOM2, MYOZ1, MYOZ2, MYPN, NEXN, NNT, OBSCN, PDLIM3, PKP2, PLEC, PLN, PRDM16, PRKAG2, RBM20, RYR2, SCN5A, SGCD, SGCG, SLC25A4, SNTA1, TAZ, TCAP, TMEM43, TNNC1, TNNI3, TNNT2, TPM1, TTN, TTR, VCL.*

**Table S1.** Criteria for classifying pathogenic variants according to ACMG guideline

| Evidence of pathogenicity | | Category |
| --- | --- | --- |
| Very strong | PVS1 | null variant (nonsense, frameshift, canonical ±1 or 2 splice sites, initiation codon, single or multiexon deletion) in a gene where loss-of-function is a known mechanism of disease |
| Strong | PS1 | Same amino acid change as a previously established pathogenic variant regardless of nucleotide change |
|  | PS2 | De novo (both maternity and paternity confirmed) in a patient with the disease and no family history |
|  | PS3 | Well-established in vitro or in vivo functional studies supportive of a damaging effect on the gene or gene product |
|  | PS4 | The prevalence of the variant in affected individuals is significantly increased compared with the prevalence in controls |
| Moderate   | PM1 | Located in a mutational hot spot and/or critical and well-established functional domain without benign variation |
|  | PM2 | Absent from controls (or at extremely low frequency if recessive) in Exome Sequencing Project, 1000 Genomes Project, or Exome Aggregation Consortium |
|  | PM3 | For recessive disorders, detected in trans with a pathogenic variant |
|  | PM4 | Protein length changes as a result of in-frame deletions/insertions in a nonrepeat region or stop-loss variants |
|  | PM5 | Novel missense change at an amino acid residue where a different missense change determined to be pathogenic has been seen before |
|  | PM6 | Assumed de novo, but without confirmation of paternity and maternity |
| Supporting   | PP1 | Cosegregation with disease in multiple affected family members in a gene definitively known to cause the disease |
|  | PP2 | Missense variant in a gene that has a low rate of benign missense variation and in which missense variants are a common mechanism of disease |
|  | PP3 | Multiple lines of computational evidence support a deleterious effect on the gene or gene product |
|  | PP4 | Patient’s phenotype or family history is highly specific for a disease with a single genetic etiology |
|  | PP5 | Reputable source recently reports variant as pathogenic, but the evidence is not available to the laboratory to perform an independent evaluation |

Adapted by permission from Springer Nature: Genetics in Medicine (1) Standards and guidelines for the interpretation of sequence variants: a joint consensus recommendation of the American College pf Medical Genetics and Genomics and the Association for Molecular Pathology. Richards et al.

PVS, very strong; PS, strong; PM, moderate; PP, supporting.

**Table S2.** **Rules for combining criteria for pathogenic and likely pathogenic variants**

| Pathogenic | (i) 1 Very strong (PVS1) AND (a) ≥1 Strong (PS1–PS4) OR (b) ≥2 Moderate (PM1–PM6) OR (c) 1 Moderate (PM1–PM6) and 1 supporting (PP1–PP5) OR (d) ≥2 Supporting (PP1–PP5) |
| --- | --- |
|  | (ii) ≥2 Strong (PS1–PS4) |
|  | (iii) 1 Strong (PS1–PS4) AND (a)≥3 Moderate (PM1–PM6) OR (b)2 Moderate (PM1–PM6) AND ≥2 Supporting (PP1–PP5) OR (c)1 Moderate (PM1–PM6) AND≥4 supporting (PP1–PP5) |
| Likely pathogenic   | (i) 1 Very strong (PVS1) AND 1 moderate (PM1–PM6) |
|  | (ii) 1 Strong (PS1–PS4) AND 1–2 moderate (PM1–PM6) |
|  | (iii) 1 Strong (PS1–PS4) AND ≥2 supporting (PP1–PP5) |
|  | (iv) ≥3 Moderate (PM1–PM6) |
|  | (v) 2 Moderate (PM1–PM6) AND ≥2 supporting (PP1–PP5) |
|  | (vi) 1 Moderate (PM1–PM6) AND ≥4 supporting (PP1–PP5) |

Adapted by permission from Springer Nature: Genetics in Medicine (1) Standards and guidelines for the interpretation of sequence variants: a joint consensus recommendation of the American College pf Medical Genetics and Genomics and the Association for Molecular Pathology. Richards et al.

PVS, very strong; PS, strong; PM, moderate; PP, supporting.

**Table S3. Primers for Sanger sequencing confirmation**

|  | Forward primer | Reverse primer |
| --- | --- | --- |
| ACTC1-1 | ACTTAATTGATTTCTTACCGT | GTGTCAACTCAGGGTTAAATG |
| ACTC1-2 | TACGGCCAGAAGCATACAGGG | CTTGACTTGGGCAGTTAGATA |
| DMD | ATATTTATGGGGTTATTACTA | CGAGCAGGGTCCAATTGTATC |
| DSC2 | GCAACCTTGCATCTAGCCATA | GGCCTCATTTCAACATTGTTC |
| DSP-1 | CCCGGACCTGCGCTACGAGGT | CGGGAAGTTCTTTCGGGACCT |
| DSP-2 | CATAGATTTGCAACCTTGCCA | CTGGAGCCCCTTCAGGTATGC |
| DSP-3 | ACAGAACGCTCCCGATATCAG | GCAGATGCTCCAGCGATAGAT |
| LAMP2-1 | GCCATTACGAGCTTGTTATGC | GAGGGACACAGCAATATCAAA |
| LAMP2-2 | TGTTCCGGTTGCAGAGTATAT | TATGCCCTTTAAAATGATAAT |
| LMNA | CCGAGATCGCGCCACTACACT | AAACAAACAGAAGCGCCACAA |
| MYBPC3-1 | ATGTGCAGCACCTCAACTGGC | ACCTCCAGTGGGGGGCTCTGC |
| MYBPC3-2 | CAGGATCCATTCGGCATTATA | GCTAACAGGATCCCGAAACTC |
| MYBPC3-3 | CTGGAATGGGAGTGGGTTCAA | GGATTACAGGCGTCTGGCCTT |
| MYBPC3-4 | ACATTATATTCTTTCGAGGAG | TGGTGCTCAGGCAATTATGTA |
| MYH7-2 | GGGTCCCAACTCACATCGAAG | AGTGGGCAATGAGTACGTCAC |
| MYH7-3 | TCTCTGTCCACCCAGGTGTAC | GGAAGGGACTCACTGGTAACT |
| MYH7-4 | GAGAAAGACACCTAGCCATG | GACCGTCCGGAACGACAACTC |
| MYH7-5 | GAGGAGGAATAGCAGTTGAAG | GACCAAGAACCCACCAATTCC |
| MYH7-6 | GAGCAAGGTCAGCAAGGGTCC | CTCTTGCTGGGCTCCTTAATG |
| KCNE1-1 | TCATGGGGAAGGCTTCGTCTC | AGCAGGGTGGCAACATGTCGG |
| KCNE1-2 | GTGTGTTGGGTTGTTCTATGG | AGCTGCAGCAGTGGAACCTTA |
| NNT | AGAGAATGCTGGACATGTTCA | AAGAGAATGCTCAGTTTGACC |
| SCN5A | CCTGCCTCAGCCTTCCGAGTA | CCCCACTCCCTACAAGCTTTA |
| TNNT2 | TTCCCAGTAATTATATCACAT | TGTCCTGACTTCTAACACCGT |
| TPM1 | AGTCACAGGGGCAGGACTGAT | CCCCCACCCAGCAATATTAGA |
| TTN-1 | GGCGTTCCACTTGTAGGTGA | GAGACTCCTGGAAAGGCCAC |
| TTN-2 | GTTACTGGACCTGGCCTTCC | CCTGCTCCACCTAGGAGACT |
| TTN-3 | ATTAACGGCCACAGACCGAG | GTGAACCAGTCCCTGCAAGA |
| TTN-4 | AGGAGGTTGTGGCACTTCTG | TGTGAGAGTTCTGGACACGC |
| TTN-5 | TGGAAGGGGTTTGCCAAGAA | CCAAGCCTACCATCAGAGCC |
| TTN-6 | AAGGCAAGCTTGGTTCTCCA | AAAATAGGCACAGGGCCTCC |
| TTN-7 | AGGTTTTCAGGCTCACCTGG | GGTCCGAGAAAAGAGGGTGG |
| TTN-8 | AGGTTTTCAGGCTCACCTGG | GCACAGCACAATGGAACAGG |
| TTN-9 | TACCGGCTGCATTGGAAACT | TTGAAAAGATCCCCCAGGGC |
| TTN-10 | TGAAGGCTTGCTGACTCCTG | GTATTGGCCCACCTGTGGAA |
| TTN-11 | TTTCAACAGGAGGGCCACAG | GGGGAGCTGGATAAAGACCG |
| TTN-12 | AGACTGGGCCAAACATACCA | GAACCAGTTCAGGCCTCTCC |
| TTN-13 | TGACAAAGGAGATGAGGTTGC | CTGCAGAGCCAGAAGTTCCA |
| TTN-14 | CCAACAGGGCAGTAAGGGAA | AAGGGGTTGCTTCAGCTGTT |
| TTN-15 | AGCATCTGAGGGGGAGATGT | TTGGATCCCAGGTTCCCCTA |

**Table S4. Pathogenic and likely pathogenic variants detected in the cohort.**

| **Gene** | **dbSNP** | **Variant type** | **Transcript** | **Transcript effect** | **Protein effect** | **Novel variant** | **Pathog enicity*** | **Evidenc e†** | **gnomAD_ ALL‡** | **gnomAD _EAS§** | **Carriers ID** |
| --- | --- | --- | --- | --- | --- | --- | --- | --- | --- | --- | --- |
| ***ACTC1*** | rs193922680 | missense | NM_005159 | c.G301A | p.E101K |  | P | PS1,PS3,PM2,PP5 | 0.00000406 | NA | 7 |
| ***ACTN2*** |  | frameshift deletion | NM_001278344 | c.89delA | p.D30fs | Novel | LP | PVS1,PM2 | NA | NA | 2 |
| ***DMD*** | rs1800278 | missense | NM_000109 | c.A547G | p.N183D | Novel | P | PS1,PS3,PP5 | NA | NA | 9 |
|  | rs41305353 | missense | NM_000109 | c.A542T | p.E181V | Novel | P | PS1,PS3,PP5 | NA | NA | 9 |
|  | rs1800278 | missense | NM_004014 | c.A547G | p.N183D | Novel | P | PS1,PS3,PP5 | NA | NA | 19 |
|  | rs41305353 | missense | NM_004014 | c.A547T | p.E181V | Novel | P | PS1,PS3,PP5 | NA | NA | 19 |
| ***DSP*** |  | frameshift insertion | NM_001008844 | c.1_2insC | p.M1fs | Novel | LP | PVS1,PM2 | NA | NA | 26,27 |
| ***KCNE1*** | rs79654911 | missense | NM_000219 | c.G200A | P.R67H |  | P | PS1,PS3,PM2,PP3,PP5 | 0.00005774 | 0.00005299 | 2 |
|  | rs1805128 | missense | NM_000219 | c.G253A | p.D85N | Novel | P | PS1,PS3,PP5 | NA | NA | 16 |
| ***MYBPC3*** |  | frameshift deletion | NM_000256 | c.1352_1379del | p.E451fs | Novel | LP | PVS1,PM2 | NA | NA | 18 |
|  |  | frameshift deletion | NM_000256 | c.2568delG | p.R856fs | Novel | LP | PVS1,PM2 | NA | NA | 26 |
| ***MYH7*** | rs121913637 | missense | NM_000257 | c.C2155T | p.R719W |  | P | PS1,PS3,PM2,PP5 | 0.00003231 | NA | 19 |
| ***NNT*** |  | frameshift insertion | NM_012343 | c.1770dupC | p.D590fs | Novel | LP | PVS1,PM2 | NA | NA | 19 |
| ***TTN*** |  | stop-gain | NM_001256850 | c.13141 | NA | Novel | LP | PVS1,PM2 | NA | NA | 9 |
|  |  | stop-gain | NM_001256850 | c.C49821G | p.Y16607X | Novel | LP | PVS1,PM2 | NA | NA | 10 |
|  |  | stop-gain | NM_001256850 | c.C34667A | p.S11556X | Novel | LP | PVS1,PM2 | NA | NA | 14 |
|  |  | frameshift deletion | NM_001256850 | c.75863delA | p.H25288fs | Novel | LP | PVS1,PM2 | NA | NA | 16 |
|  |  | stop-gain | NM_001256850 | c.C22335A | p.Y7445X | Novel | LP | PVS1,PM2 | NA | NA | 28 |

* Determined according to criteria in Table S2

† As listed in Table S1

‡ Minor allele frequencies of variants among total population in the Genome Aggregation Database (2)

§Minor allele frequencies of variants among East Asians in the Genome Aggregation Database(2)

LP, likely pathogenic; NA, not available; P, pathogenic

**Table S5. Left ventricular segmental strain data assessed by CMR feature tracking**

| **Parameters** | **LVNC(n=28)** | **G+ (n=11)** | **G- (n=17)** | ***p* value** |
| --- | --- | --- | --- | --- |
| Basal LS, % | -15.9(-18.2, -10.2) | -14.9(17.3, -7.1) | -15.9(-19.8, -10.7) | 0.557 |
| Mid LS, % | -14.1(-17.5, -11.0) | -13.2(-17.6, -9.7) | -14.1(-17.9, -12.4) | 0.655 |
| Apical LS, % | -11.2(-16.7, -5.9) | -11.0(-15.0, -5.7) | -11.9(-18.3, -5.9) | 0.495 |
| Basal CS, % | -17.5(-27.3, -9.6) | -17.0(-21.9, -9.5) | -18.9(-29.5, -10.4) | 0.335 |
| Mid CS, % | -11.2(-21.6, -6.1) | -10.9(-17.0, -4.6) | -15.7(-21.8, 6.6) | 0.495 |
| Apical CS, % | -21.3(-29.2, -10.4) | -14.8(-33.5, -5.7) | -25.0(-28.7. -12.8) | 0.269 |
| Basal RS, % | 18.4(9.1, 36.2) | 26.5(10.3, 29.1) | 11.6(8.5, 45.4) | 0.495 |
| Mid RS, % | 21.2(9.0, 43.3) | 17.0(3.1, 45.1) | 22.3(11.0, 42.9) | 0.796 |
| Apical RS, % | 32.5(14.4, 52.9) | 20.7(11.0, 35.4) | 42.4(21.1, 55.3) | 0.151 |

Data are expressed as median (inter-quartile range).

*LVNC* left ventricular non-compaction cardiomyopathy, *G+* genotype positive, *G-* genotype negative, *LS* longitudinal strain, *CS* circumferential strain, *RS* radial strain.

**Table S6. Univariate Cox analysis of LV segmental strain for predicting primary endpoint**

| **Parameters** | **LR Chi2 (*P* Value)** | **Wald** | **HR (95% CI)** | ***P* Value** |
| --- | --- | --- | --- | --- |
| Basal LS | 6.854(0.009) | 6. 021 | 1.157(1.030-1.300) | 0.014 |
| Mid LS | 4.382(0.036) | 4.632 | 1.138(1.012-1.280) | 0.031 |
| Apical LS | 4.045(0.044) | 3.750 | 1.108(0.999-1.230) | 0.053 |
| Basal CS | 11.675(0.001) | 8.854 | 1.169(1.055-1.296) | 0.003 |
| Mid CS | 12.106(0.001) | 8.426 | 1.238(1.072-1.429) | 0.004 |
| Apical CS | 5.029(0.025) | 4.557 | 1.074(1.006-1.148) | 0.033 |
| Basal RS | 3.110(0.078) | 2.701 | 0.962(0.919-1.007) | 0.100 |
| Mid RS | 5.165(0.023) | 4.975 | 0.967(0.938-0.996) | 0.026 |
| Apical RS | 1.556(0.208) | 1.582 | 0.979(0.948-1.012) | 0.208 |

*LS* longitudinal strain, *CS* circumferential strain, *RS* radial strain.

1. Richards S, Aziz N, Bale S, Bick D, Das S, Gastier-Foster J, Grody WW, Hegde M, Lyon E, Spector E, Voelkerding K, Rehm HL. Standards and guidelines for the interpretation of sequence variants: a joint consensus recommendation of the American College of Medical Genetics and Genomics and the Association for Molecular Pathology. Genet Med 2015;17(5):405-424. doi: 10.1038/gim.2015.30

2. Lek M, Karczewski KJ, Minikel EV, Samocha KE, Banks E, Fennell T, O'Donnell-Luria AH, Ware JS, Hill AJ, Cummings BB, Tukiainen T, Birnbaum DP, Kosmicki JA, Duncan LE, Estrada K, Zhao F, Zou J, Pierce-Hoffman E, Berghout J, Cooper DN, Deflaux N, DePristo M, Do R, Flannick J, Fromer M, Gauthier L, Goldstein J, Gupta N, Howrigan D, Kiezun A, Kurki MI, Moonshine AL, Natarajan P, Orozco L, Peloso GM, Poplin R, Rivas MA, Ruano-Rubio V, Rose SA, Ruderfer DM, Shakir K, Stenson PD, Stevens C, Thomas BP, Tiao G, Tusie-Luna MT, Weisburd B, Won HH, Yu D, Altshuler DM, Ardissino D, Boehnke M, Danesh J, Donnelly S, Elosua R, Florez JC, Gabriel SB, Getz G, Glatt SJ, Hultman CM, Kathiresan S, Laakso M, McCarroll S, McCarthy MI, McGovern D, McPherson R, Neale BM, Palotie A, Purcell SM, Saleheen D, Scharf JM, Sklar P, Sullivan PF, Tuomilehto J, Tsuang MT, Watkins HC, Wilson JG, Daly MJ, MacArthur DG. Analysis of protein-coding genetic variation in 60,706 humans. Nature 2016;536(7616):285-291. doi: 10.1038/nature19057
